# Cognitive behavioral therapy for adults with attention-deficit hyperactivity disorder: study protocol for a randomized controlled trial

**DOI:** 10.1186/s13063-015-0686-1

**Published:** 2015-04-14

**Authors:** Fang Huang, Qiujin Qian, Yufeng Wang

**Affiliations:** Peking University Sixth Hospital/Institute of Mental Health, National Clinical Research Center for Mental Disorders (Peking University Sixth Hospital), No. 51, Hua Yuan Bei Lu, Haidian District, Beijing, 100191 China; Key Laboratory of Mental Health, Ministry of Health, Peking University, No. 51, Hua Yuan Bei Lu, Haidian District, Beijing, 100191 China

**Keywords:** Attention-deficit hyperactivity disorder (ADHD), cognitive behavioral therapy (CBT), booster sessions, randomized controlled trial

## Abstract

**Background:**

Attention-deficit hyperactivity disorder (ADHD) is a mental disorder beginning in childhood, and about half of patients have symptoms lasting into adulthood. Adult ADHD causes various impairments of emotional, self-esteem, and executive function and life quality aspects. Furthermore, adverse outcomes include academic and occupational failures, traffic accidents and substance abuse, which would be a family and social burden. A combination of medication and psychotherapy is recommended as the treatment for adult ADHD, and cognitive behavioral therapy (CBT) has been validated mostly with evidence-based researches. However, there has been a lack of randomized controlled trials of CBT for patients in China. Moreover, booster sessions of CBT for other disorders have proven effective in reducing recurrence and improving long-term outcomes, which has not been investigated for adult ADHD. This study will testify to the effect of CBT and explore the efficacy of subsequent booster sessions on adult ADHD.

**Methods/Design:**

It is a three-armed randomized controlled trial to evaluate the efficacy of 12 weeks of CBT based on the published and validated manual and its booster sessions. The 12 weeks of CBT will be conducted weekly and will end at the 12th week, and then the booster sessions will be conducted monthly and end at the 24th week. There are three randomized groups, including a CBT with booster sessions group, a CBT group and a waiting group. Participants are outpatients of the Peking University Sixth Hospital who are diagnosed as having adult ADHD. The Primary efficacy endpoints are the scores of ADHD core symptoms at 12 and 24 weeks. Secondary endpoints include emotion, executive function, self-esteem, life quality and functional magnetic resonance imaging (fMRI) data at different time points, and the change within every group will also be analyzed.

**Discussion:**

This is the first study to explore the efficacy of booster sessions of CBT in adult ADHD as far as we know. The results might increase proof of efficacy of CBT for adult ADHD in China, and the results showing efficacy of the booster sessions would also benefit our clinical practice.

**Trial registration:**

Current Controlled Trials: NCT02062411, date of registration: 12 February 2014.

## Background

Attention-deficit hyperactivity disorder (ADHD) begins in childhood [1], and 50% of patients have symptoms lasting into adulthood [2]. The prevalence of adult ADHD is about 5% [3]. There are different adverse outcomes along with adult ADHD including comorbidities as depression and anxiety; cognitive function impairment; social dysfunctions such as procrastination, dropping out of school, and being laid off; increasing rates of traffic accidents; substance abuse; and decreases in quality of life [4-8]. This disease could cause a great burden of society, and the human capital value was reported as $4,336 per worker for a single year in the United States [9]. The clinical treatment guideline recommends a combination of medication and psychotherapy as the first line therapy method for adult ADHD [10,11], and the cognitive behavioral therapy (CBT) has been validated mostly with evidence-based researches [12-15].

The efficacy of CBT in adult ADHD has been proven in several randomized studies on the aspects of core symptoms, emotion, self-esteem and time-management strategies [12-15]. Studies also found the efficacy of CBT was superior to relaxation with educational support [13] and computerized cognitive training [16]. Compared with medication, CBT could also improve patients’ ADHD symptoms without previous medical treatment, which might suggest that the mechanism of CBT did not fully rely on medication [14]. However, the previous studies mainly focused on some selected aspects of CBT’s efficacy, and the multidimensional impairments that proved to be prominent for adult ADHD patients [4-8] had not been tested in the same group of subjects, which left the measurement of CBT’s efficacy remaining to be explored [17]. Our group’s self-control study also found preliminary evidence of the efficacy of 12 weeks of CBT in a Chinese population [18,19], which needs to be further validated in a randomized controlled study.

A review of CBT studies for adult ADHD emphasizes that evidence of long-term outcomes is limited [17]. Through the follow-up of treated patients from our group study, which only used 12 weeks of CBT as described in the manual [18], we found that patients forgot the skills they learned in CBT and had difficulties in applying them into practice in real life. The booster sessions were previously used in behavior therapy to achieve sustaining results, and a review of 30 clinical trials concluded that the booster sessions were useful in maintaining behavioral change [20]. With regard to CBT, according to studies covering depression [21,22], bipolar disorder [23], panic disorder [24] and binge eating disorder [25], providing the booster sessions or the maintaining treatment after the completion of CBT would reduce the recurrence rate and benefit the long-term effect. Furthermore, the latest meta-analyses found that CBT with booster sessions was more effective and had more sustainable outcomes for children with mood or anxiety disorders [26]. To date, three CBT studies for adult ADHD have included periods as booster sessions [13,14,27]; however, the specific effects of this process have not been investigated. In Safren’s study, there is an optional session that contains review and summary of the previous lessons [13]. There were two booster sessions in Weiss’s research at 15 and 20 weeks [14], and 10 booster sessions monthly in Philipsen’s study protocol after 12 weeks of group therapy [27]. None of these above studies considered these periods as separate from the treatment or explored their role in the integrated treatment.

The integration of known CBT interventions for other disorders in adult ADHD remain to be explored [17]. So, we wonder whether the booster sessions could help CBT in fulfilling the skill-teaching and practicing missions, thereby prolonging the efficacy for adults with ADHD. This study is conducted to test whether CBT is effective in the treatment of attention-deficit and emotional, executive and social impairments due to ADHD in a randomized controlled design and to explore the role booster sessions played in the CBT process.

### Objectives/hypotheses

The primary research questions are as follow: (1) Is CBT effective in the treatment of adult ADHD and related problems? and (2) Is the treatment effect of CBT with booster sessions superior to that of CBT only?

There are still other questions: (1) Would booster sessions improve the patients’ satisfaction at evaluation? The subjective evaluation would reflect the acceptability of the treatment, especially in psychotherapy, and these measurements might reflect factors that work other than outcomes of scales or behavioral tests [15,28]. (2) Are there some significant factors that can predict or influence the outcome, including symptom severity, comorbidity, social and economic status, and neuropsychological or neuroimaging biological markers? The role of baseline situations in CBT for adult ADHD remains unclear, and the possible biological markers might benefit finding the “working factors” in CBT [17,29,30]. (3) Would the effects of CBT cause some neuroimaging changes? The neuroimaging methods would provide the biological evidence of treatment and benefit exploring the mechanism of psychotherapy [29]. (4) Finally, considering the limited evidence of long-term effects [17,30], how long the outcomes last would also be evaluated.

The hypothesis of this study is that CBT reduces ADHD symptoms compared to nonintervention, and CBT with booster sessions reduces more ADHD symptoms compared to CBT only.

## Methods/Design

### Study design

We use a three-armed study design in order to evaluate the effect of CBT on ADHD adults compared with no intervention condition and the effect of 12 weeks CBT with booster sessions versus 12 weeks CBT only. There are three randomized groups, including a CBT with booster sessions group (group 1), a CBT group (group 2) and a waiting group (group 3).

**Figure 1:**
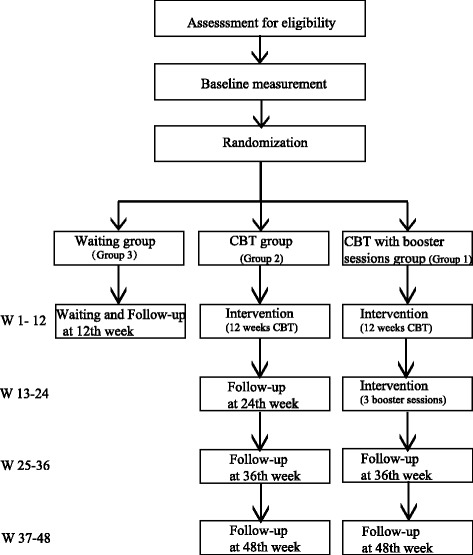
Trial flow. W, week; CBT, cognitive behavioral therapy.

The primary and secondary outcomes will be evaluated at baseline (T1), after 12 weeks of treatment (T2) and after three booster sessions, which is at 24 weeks (T3). Moreover, two ADHD symptom evaluation scales and two emotion evaluation scales will be rated weekly during the 12 weeks of treatment in order to monitor the changes. Patients of the CBT with booster sessions group (group 1) will be followed up at 36 weeks and 48 weeks. Furthermore, the follow-up will be conducted at 24 weeks, 36 weeks and 48 weeks for patients of the CBT group (group 2), and will be at 12 weeks for the waiting group (group 3) patients (Figure 1). Considering the interest of the waiting group patients, on request, we will provide them with 12 weeks of CBT at no charge after the study.

This study is conducted in the Peking University Sixth Hospital and funded by Beijing Municipal Science and Technology Commission. The research is based on good clinical practice standards (GCP) and the CONSORT statement [31,32], which received ethics approval from Ethics Committee of the Sixth Hospital of Peking University ((2013) Ethics review number (42)). It has been registered in ClinicalTrials.gov (NCT02062411).

### Intervention

In this study, 12 weeks of CBT is being conducted according to a published and validated [12,13] manual (Mastering Your Adult ADHD: A Cognitive-Behavioral Therapy Approach, Safren SA, Perlman CA, Sprich S, Otto MW, 2005) [33]. The original manual consisted of three core modules and two optional modules, and we put all five modules into practice. The first part (five sessions) consists of psychoeducation; supportive relationships; and organizational skills such as using a calendar system, breaking tasks into small steps and evaluating the benefits and shortcomings of choices. The second part (two sessions) is mainly about coping with distractibility, including the skill to record distractive thoughts and management of the surroundings. The third part (three sessions) is cognitive restructuring, which helps patients identify the maladjusted automatic thoughts and change them into adaptive ones. The last part (two sessions) includes dealing with procrastination with all the learned skills and summary. All the 12 sessions are conducted once a week at weekends. The English manual was translated into Chinese by our group’s senior psychiatrists who received psychotherapy training, and the Chinese edition was applied to two different groups of patients in self-control studies, which was effective for reducing ADHD symptoms and improving everyday life executive function, self-esteem and life quality [18,19].

The booster sessions are designed according to the definition of booster sessions in studies of ADHD [13,14,27] and other diseases [21-25], the main goal of which is consolidating the skills learned and developing strategies dealing with recurrence or relapse. Thus, the booster sessions in the present study contain the induction of the first three parts above and practicing activities based on the patients’ questions in the real world. The first booster session includes a summary of the first organizational part of the 12-week manual. It also includes other important time-management skills such as goal setting, reward system and authorizing others [34], and a discussion of the practice questions through role-play. The second booster session consists of the summary of the distractibility part, the discussion of the relationship between distractibility and procrastination, and making a self-coping list. The last booster session includes a summary of the cognition part, the discussion of automatic thoughts of real situation by role-play, and the relationship between automatic thoughts and procrastination. These three booster sessions are conducted monthly after the complement of 12 weeks of CBT.

### Participants

The subjects mainly are outpatients of the Peking University Sixth Hospital. Patients who read the recruitment on the internet, come to the outpatient service and meet the inclusion criteria will also be included. All subjects will be fully informed about the research before being asked to sign the informed consent form.

Patients with adult ADHD are recruited. With regard to the former treatment, we include people who have been stable on medications for adult ADHD for at least 2 months (drug dose was adjusted within 10% in last two months [13]). The key inclusion criteria include the following:

1. Outpatients from Peking University Sixth Hospital, who have received a diagnosis of adult ADHD through Conners’ Adult ADHD Diagnostic Interview [35] based on Diagnostic and statistical manual of mental disorders, Fourth Edition (DSM-IV).
2. Patients who are either medication naive or have been stable on medications (for the treatment of ADHD, drug dosage adjustment in last two months was below 10% [12]) for adult ADHD for at least 2 months.

Furthermore, patients with comorbidity that may influence the study outcomes or need extra treatment are excluded. Patients who are at risk for suicide, in unstable physical condition, or have received psychological treatment will be excluded. To make some cognition function assessments possible, people who are older than 45 years old or have intelligence quotient (IQ) less than 90 will be excluded. The key exclusion criteria include the following:

1. Severe major depression, clinically significant panic disorder, bipolar disorder, organic mental disorders, psychotic disorders, or pervasive developmental disorders
2. Age older than 45, which might influence outcomes of cognitive tests [36] or IQ less than 90 [12]
3. Suicide risk
4. Unstable physical condition, which needs medical treatment prior to ADHD such as active hepatitis and angina pectoris
5. Prior or present participation in other psychological therapies.

### Outcome measures

The primary efficacy endpoint is an ADHD Rating Scale (ADHD-RS) [37] score at 12 and 24 weeks. We will measure the different scores between the waiting group (group 3) and the CBT group (group 2) at 12 weeks to prove the effect of 12 weeks CBT on adult ADHD, and between the CBT with booster sessions (group 1) and the CBT group (group 2) at 24 weeks to explore the effect of booster sessions.

The main secondary endpoints will include the following:

- ADHD symptoms (Conners Adult ADHD Rating Scale Self-report Screening Version [38]) at 2, 3, 4, 5, 6, 7, 8, 9, 10, 11, 36, 48 weeks
- Emotional symptomatology (Self-rating Anxiety Scale [39], Self-rating Depression Scale [40]) at 2, 3, 4, 5, 6, 7, 8, 9, 10, 11, 12, 24, 36, 48 weeks
- Executive function (Behavior Rating Inventory of Executive Function-Adult Version (BRIEF-A) [41], Cambridge Neuropsychological Testing Automated Battery (CANTAB) [42] at 12, 24, 48 weeks
- Impulsiveness (Barratt impulsiveness scale [43]) at 12, 24, 48 weeks
- Self-esteem (self-esteem scale [44]) at 12, 24, 48 weeks
- Life quality (World Health Organization Quality of Life-Brief Version [45]) at 12, 24, 48 weeks
- Neuroimaging (Brain Oxygenation Level Dependent Signal) at 12 weeks.

We summarized the tools and the assessing time points in Table 1. The reasons we used these instruments to measure the efficacy are explained below. Since the ADHD symptoms are the main target of treatment and this manual was specifically designed for ADHD, the measurement of core ADHD symptoms is significant for confirming the effect of CBT. And the level of impulsiveness may manifest the core impairment of ADHD [46]. It was reported that emotional disorders were common for adult ADHD as comorbidities (the lifetime comorbid rates were 19.4% for generalized anxiety disorder and 28% for major depression [47]) and 32% of ADHD adults had emotional dysregulation [48], which makes it important to monitor the change of emotion during the intervention. Furthermore, the latest meta-analysis proved that CBT was effective for depression and anxiety [49], so the emotional results would be positive. The executive function of ADHD adults was reported to be impaired [50], and the BRIEF-A and the CANTAB could test both the ecological and practical aspect [51]. Moreover, the levels of self-esteem and life quality of ADHD adults were also proven to be lower than those of normal controls [52,53]. Additionally, the neuroimaging method as well as the neuropsychological tests could help identify the potential endophenotypic biomarkers that reflect or predict the outcomes of CBT, which would provide a strong evidence of the efficacy of psychotherapy [29]. Given the main consideration of core symptoms and the relatively great change of emotion [12,18,19], these were tested frequently. Furthermore, other aspects were evaluated, as done in previous studies [12-16,27], at three main endpoints, which were before treatment, after CBT and after booster sessions, whereas imaging was conducted at the end of 12 weeks because the efficacy of 12 weeks of CBT on brain functions had not been tested in adult ADHD as had been done in other studies [54,55].

**Table 1:** **Instruments applied at different time points**

**Instruments**	**Baseline**	**Week 2 to 11**	**Week 12**	**Week 24**	**Week 36**	**Week 48**
ADHD-RS	×	×	×	×	×	×
CAARS	×	×	×	×	×	×
SAS	×	×	×	×	×	×
SDS	×	×	×	×	×	×
BRIEF-A	×		×	×		×
CANTAB	×		×			
BIS	×		×	×		×
SES	×		×	×		×
WHOQOL-BRIEF	×		×	×		×
fMRI	×		×			

ADHD-RS, ADHD Rating Scale; CAARS, Conners Adult ADHD Rating Scale Self-report Screening Version; SAS, Self-rating Anxiety Scale; SDS, Self-rating Depression Scale; BRIEF-A, Behavior Rating Inventory of Executive Function-Adult Version; CANTAB, Cambridge Neuropsychological Test Automatic Battery; BIS, Barratt impulsiveness scale; SES, self-esteem scale; WHOQOL-BRIEF, World Health Organization Quality of Life-Brief Version; fMRI, functional magnetic resonance imaging.

### Blinding and quality control

There is an independent statistician who conducts the randomization of patients and analyzes the research results. The participants and endpoint assessors are kept blind during the study. Only the principal investigator is informed of the randomization results at first, and the psychotherapists are told when the 12 weeks of CBT is completed.

There are two psychiatrists who conduct the CBT and they have received same systematic psychotherapy training, including CBT training. They received psychological training courses held by the Peking University Sixth Hospital and the Chinese Psychological Society. One psychiatrist is in charge of the therapy, and the other is the recorder who writes down all the sessions. There also are two supervisors, including a psychiatrist and a psychotherapist. Regular discussion and supervision are conducted during the study. The assessors are mainly postgraduate students of psychiatry, and they are trained to use all the measurement tools and the consistency is rated.

The principal investigator monitors the research progress and ensures all the steps are taken according the original protocol, and the Ethics Committee supervises the interests of subjects such as safety and confidentiality.

### Power

It is a noninferiority randomized controlled study, and the primary outcome measure is the mean difference of the ADHD-RS scores between groups. Since there was no relevant study that included booster sessions for adult ADHD, we used the results of a self-controlled preliminary experimental study. At baseline, the score of the ADHD rating scale was 29.80 ± 7.67 (n = 15), after CBT the score was 17.22 ± 7.62 (n = 18), and after booster sessions the score was 10.56 ± 4.98 (n = 16). We assume the drop-out rate is 10%, because the previous studies showed good compliance, and the outpatients are willing to participate [18,19]. So with the power of 80% and alpha of 5% and based on the formula (n = 2[(tα + tβ)*σ/δ]^2, n means number of subjects, tα means t value of alpha, tβ means t value of beta, σ means population standard deviation, δ means deviation), the sample size is about 73 subjects including 15 patients in the waiting group (group 3), 29 in the CBT group (group 2), and 29 in the CBT with booster sessions group (group 1). The numbers of subjects varied in the three groups since the deviations (δ) of ADHD-RS scores after 12 week CBT and after booster sessions were different. Considering the real situation with recruitment, we separated it into three batches, with a total of 21, 24 and 28 subjects for one time (Table 2).

**Table 2:** **Numbers of subjects in three batches of the study**

**Batch**	**Group 1**	**Group 2**	**Group 3**	**Total**
1	7	7	7	21
2	8	8	8	24
3	14	14	-	28
Total	29	29	15	73

Group 1: Cognitive behavioral therapy (CBT) with booster sessions group; Group 2: CBT group; Group 3: waiting group.

### Statistical analysis

The main analysis is a two-group ANCOVA of ADHD symptoms, emotional symptomatology, executive function, impulsiveness, self-esteem and life quality at all time points with baseline scores as covariates. To estimate the changes with time in every group, the repeated measures ANOVA will also be used. Furthermore, the neuroimaging data will be analyzed based on a voxel-by-voxel method and the general linear model with a one group by two time points design after pre-processing procedures. The statistics is based on intent-to-treat (ITT) analysis, and the last observation carried forward (LOCF) will be used to address the missing data.

The primary endpoints analysis is ANCOVA with ADHD-RS scores at 12 and 24 weeks separately as dependent variables and baseline ADHD-RS scores as covariates. Other variables that have correlation with ADHD-RS scores and clinical meanings will also be considered for covariates such as gender, age, IQ and education.

## Discussion

This is the first study to explore the efficacy of booster sessions of CBT in adult ADHD as far as we know. In this study, we hypothesize that the 12 weeks of CBT will affect core symptoms and other functions for patients compared to waiting group, and that the booster sessions could improve and prolong these effects compared with patients who receive CBT with no booster sessions.

### Strengths

This study has several strengths: (1) the randomized controlled design with three arms benefits the efficacy validation of using both 12 weeks of CBT and booster sessions in one study, which could be cost and time saving; (2) standardized diagnostic and assessment instruments including subjective self-rating scales, and objective computer-based (CANTAB) and neuroimaging evaluations; (3) the blind statistician and the same two psychotherapists who have received regular training and supervision; (4) the use of the CBT manual, which has been proven to be effective [12,13]; and (5) the protocol was designed based on the CONSORT statement, is registered online, and is under the supervision of an Ethics Committee.

### Limitations

The exclusion of several comorbidities might narrow the scope of application of the results since comorbidity is common for adult ADHD patients and may reflect a more severe condition. Furthermore, the recruitment of patients from a single research center could also influence the sample representatives.

The design of the waiting group could not fully reflect the effect of intervention by comparison since the placebo effect could also play a role. So a control group with non-directive activities, such as group discussion and psychoeducation, might be more comparable. With the limitation of staff and resources in a single center, this strategy could be used in multicenter research.

The follow-up time in this study is 48 weeks (1 year), which could be insufficient for testifying to the prolonged effect of booster sessions. As the participants are outpatients, we would get the consent of subjects to allow future contact and follow-up.

The dropping-out rate might be a little optimistic based on the data of our group’s previous studies. Since this study is funded and conducted in consecutive years, the sample could be more than we estimated.

### Direction for clinical practice and future study

In this study we aimed to explore the efficacy of CBT and its booster sessions for adult ADHD. The 12 weeks of CBT, which has proven effective abroad [12,13], needs to be validated before applying it to the Chinese population. Furthermore, we evaluated its effect with various instruments covering core symptom, emotion, executive function, self-esteem, life quality, computer-based neuropsychological tests and neuroimaging scans. With all these tools, we could identify the aspects that CBT could influence and those it could not. It might be conducive for our understanding of the efficacy and mechanism of psychotherapy, which has remained unclear up to now [29].

Moreover, the study of booster sessions might remind us to value the long-term effect [17] and the real benefit of patients. Since the effects we published in papers were only transient results for patients compared with their life, we need to switch our focus from research success to considering what patients really need. And the need of booster sessions were found through the interview with treated patients. The exploration of efficacy of the booster sessions could give us the answer as to whether adding a few more sessions could enhance and prolong the effect of previous therapy. Furthermore, if the results are positive, the method of attaching booster sessions to 12 weeks CBT could be beneficial, economical and practical.

In conclusion, future studies could explore CBT with booster sessions in a more representative sample with common comorbidities and from several centers and should include a control group to avoid the placebo effect. Such a study might further prove the efficacy of CBT for adult ADHD in China, and evidence of booster session efficacy would also benefit our clinical practice.

## Trial status

Recruitment is ongoing and began in October 2013. The first patient was randomized in October 2013. As of June 2014, 66% have been randomized, and the trial is expected to be completed in July 2016.

## Abbreviations

ADHD: attention-deficit hyperactivity disorder
ADHD-RS: ADHD Rating Scale
BRIEF-A: Behavior Rating Inventory of Executive Function-Adult Version
BIS: Barratt Impulsiveness Scale
CAARS: Conners Adult ADHD Rating Scale Self-report Screening Version
CANTAB: Cambridge Neuropsychological Testing Automated Battery
CBT: cognitive behavioral therapy
DSM-IV: Diagnostic and Statistical Manual of Mental Disorders, Fourth Edition
fMRI: functional magnetic resonance imaging
GCP: good clinical practice
ITT: Intent-to-treat (ITT) analysis
LOCF: last observation carried forward
SAS: Self-rating Anxiety Scale
SDS: Self-rating Depression Scale
SES: Self-esteem Scale
WHOQOL-BRIEF: World Health Organization Quality of Life-Brief Version
